# Statistical considerations in the design and analysis of non-inferiority trials with binary endpoints in the presence of non-adherence: a simulation study

**DOI:** 10.12688/wellcomeopenres.15636.2

**Published:** 2020-04-24

**Authors:** Yin Mo, Cherry Lim, Mavuto Mukaka, Ben S. Cooper

**Affiliations:** 1 Mahidol-Oxford Tropical Medicine Research Unit, Faculty of Tropical Medicine, Mahidol University, Bangkok, 10400, Thailand; 2 Division of Infectious Diseases, University Medicine Cluster, National University Hospital, Singapore, 119074, Singapore; 3 Department of Medicine, National University of Singapore, Singapore, 119228, Singapore; 4 Centre for Tropical Medicine and Global Health, Nuffield Department of Medicine, University of Oxford, Oxford, OX3 7BN, UK

**Keywords:** Trial methodology, non-inferiority trials, causal inference, non-adherence

## Abstract

Protocol non-adherence is common and poses unique challenges in the interpretation of trial outcomes, especially in non-inferiority trials. We performed simulations of a non-inferiority trial with a time-fixed treatment and a binary endpoint in order to: i) explore the impact of various patterns of non-adherence and analysis methods on treatment effect estimates; ii) quantify the probability of claiming non-inferiority when the experimental treatment effect is actually inferior; and iii) evaluate alternative methods such as inverse probability weighting and instrumental variable estimation. We found that the probability of concluding non-inferiority when the experimental treatment is actually inferior depends on whether non-adherence is due to confounding or non-confounding factors, and the actual treatments received by the non-adherent participants. With non-adherence, intention-to-treat analysis has a higher tendency to conclude non-inferiority when the experimental treatment is actually inferior under most patterns of non-adherence. This probability of concluding non-inferiority can be increased to as high as 0.1 from 0.025 when the adherence is relatively high at 90%. The direction of bias for the per-protocol analysis depends on the directions of influence the confounders have on adherence and probability of outcome. The inverse probability weighting approach can reduce bias but will only eliminate it if all confounders can be measured without error and are appropriately adjusted for. Instrumental variable estimation overcomes this limitation and gives unbiased estimates even when confounders are not known, but typically requires large sample sizes to achieve acceptable power. Investigators need to consider patterns of non-adherence and potential confounders in trial designs. Adjusted analysis of the per-protocol population with sensitivity analyses on confounders and other approaches, such as instrumental variable estimation, should be considered when non-compliance is anticipated. We provide an online power calculator allowing for various patterns of non-adherence using the above methods.

## Introduction

Clinical trials designed to determine whether an experimental treatment is no worse than the standard-of-care treatment by a predefined margin are known as non-inferiority trials. Though a widely adopted trial design in the medical literature, the best practices for trial design, analysis and reporting remain debated. These debates often revolve around the appropriateness of the non-inferiority margin, and consistency with historical placebo-controlled trials in the choices of standard-of-care control treatment, study population and outcomes. Non-adherence to allocated treatment, which occurs commonly in all randomized controlled trials, has also been recognized as an important contributor towards making erroneous conclusions in non-inferiority trials ^1– 3^.

The most widely used analysis strategy in all clinical trials, including the non-inferiority design, is the intention-to-treat analysis (ITT). The analysis compares individuals according to their randomly allocated treatment, regardless of what actual treatment an individual receives. Hence, ITT estimates the effect of assigning a treatment instead of the treatment effect itself. The effect of assignment and the effect of treatment will generally differ when there is non-adherence. When non-adherent individuals switch to treatments prescribed in the opposite allocation, or take up other treatments with similar efficacy as the standard-of-care, the ITT estimates tend to shift towards zero difference. This property is generally valued in the analysis of superiority trials as the demonstration of superiority becomes more difficult. In non-inferiority trials, however, it may lead to the conclusion of non-inferiority for allocating the experimental treatment when the new treatment is actually inferior in terms of treatment efficacy.

While the effect of allocation may sometimes be reflective of the ‘real world’ practice, the causal effect of treatment on the outcome is often of considerable interest. This is the focus of this paper. To estimate treatment efficacy, a widely used method is the per protocol analysis (PP). This analysis considers only individuals who adhere to the allocated treatment and excludes those who do not. However, because the adherent individuals may have different characteristics compared to the non-adherent individuals in the allocation arms, comparing only the adherent individuals may lead to biased treatment estimates.

The above issues have been highlighted in international guidelines and simulation studies, but consensus on the best way forward has not been reached ^3– 6^. Of note, Kim has previously shown that the standard approaches can lead to erroneous conclusions about treatment efficacy in non-inferiority trials with non-adherence and proposed using an instrumental variable estimator as an alternative statistical method ^7^. Sanchez and Chen reached a similar conclusion: depending on the pattern of protocol deviation, both PP and ITT populations may show non-inferiority when the treatment effect is actually inferior ^8^. In the latest CONSORT guideline for non-inferiority trials, it has been suggested that hybrid ITT/PP analyses should be considered ^4^. However, the exact methodology was not specified. Practical guidance is needed when designing trials about how incremental levels of non-adherence affect the chance of reaching different trial conclusions.

It is important to assess the potential patterns of non-adherence that might occur in a non-inferiority trial during the planning stage both to inform power calculations and to allow an appropriate analysis plan to be developed ^8, 9^. However, no easily accessible tools are currently available to guide investigators in non-inferiority trial design accounting for these considerations. In this study, we performed simulations of a hypothetical non-inferiority trial with a binary outcome in order to: i) explore the impact of various patterns of non-adherence and analysis methods on trial treatment effect estimates; ii) quantify the probability of claiming non-inferiority when treatment efficacy is actually inferior; iii) compare and evaluate alternative analysis methods such as inverse probability weighting and instrumental variable estimation; and iv) provide a tool for investigators to design non-inferiority trials which anticipate non-adherence.

## Methods

We simulated a two non-inferiority randomized controlled trial, where treatment, A, and outcome, Y, are binary and time fixed. Randomization, *Z*, is done in a 1:1 ratio. An example of such a trial is the study on optimising antibiotic treatment duration for community acquired pneumonia ^10^. Theexperimental treatment is five days of antibiotic treatment ( *A* = *a*_1_), while the control treatment is a duration as decided by the physicians ( *A* = *a*_0_). Outcome is treatment failure as defined by a set of questionnaire scores on day 30 ( *Y* = 1 represents treatment failure, *Y* = 0 represents treatment success). With this single end-point, we consider adherence as a binary variable where non-adherent patients in the short arm would receive longer than five days of treatment, and non-adherent patients in the long arm would receive fewer than five days of treatment. The effect estimate is the absolute risk difference, calculated as the difference in the proportion of participants with treatment failure between treatment arms.

We calculated the sample size based on the hypothetical assumption that 40% of patients in both experimental and control arms experience treatment failure, with a non-inferiority margin of 10% and tolerable type 1 error of 0.025. This required 505 participants per arm for 90% power ^11^. We explored all simulation scenarios with 60–100% adherence to illustrate the effect of adherence under various patterns of non-adherence and analysis methods. Each simulation was performed with 1000 iterations. All simulation and analyses were performed with R Version 1.1.463 ^12^. Simulation code is available on GitHub. ( https://github.com/moyinNUHS/NItrialsimulation.git).

### Notation

In the subsequent paragraphs, *Y ^a=0^* represents the potential outcome if the control treatment were to be administered ( *A* = *a*_0_); *Y ^a=1^* represents the outcome that would occur if the experimental treatment were to be administered ( *A* = *a*_1_); and *Y ^a=2^* represents the potential outcome if an alternative inferior treatment compared to both the control and experimental treatments were to be administered ( *A* = *a*_2_). For an individual, *i*, $Y_{i}^{a\;=\;a_{0}};\;Y_{i}^{a\;=\;a_{1}};\;Y_{i}^{a\;=\;a_{2}}$ are therefore counterfactual outcomes. Because only one of the outcomes is observed in the real world, the actual observed outcome, *Y _i_*, is either equal to $Y_{i}^{a\;=\;a_{0}};\;Y_{i}^{a\;=\;a_{1}};\;Y_{i}^{a\;=\;a_{2}}$ depending on the treatment received, i.e. *Y _i_* = *Y ^A^_i_*, where *A _i_ = a*_0_ if the individual received the control treatment, *A _i_ = a*_1_ if the individual received the experimental treatment, and *A _i_ = a*_2_ if the individual received an alternative inferior treatment compared to both the control and experimental treatments ^13^. Similarly, the observed outcomes depending on randomization ( *Z*) are represented by *Y _i_^z=1^*, and *Y _i_^z=0^* respectively. *C* refers to the confounding factors that may increase or decrease the probabilities of adhering to the allocated treatment and outcome.

### Analysis methods

The ITT analysis considers all randomized participants according to their assigned arms, regardless of whether the participants had the intended treatment. It estimates the effect of *Z* on *Y,* i.e. *Pr*[ *Y* ^Z=1 ^= 1] - *Pr*[ *Y ^Z^* ^=0 ^=1]. The PP analysis only considers participants who received treatment according to their allocation stated in the study protocol, i.e. *Pr*[ *Y ^A=a^* ^_1_*,* Z=1 ^=1] - *Pr*[ *Y ^A=^* ^*a*_0_*,* Z=0^ =1].

In addition, we used an inverse probability weighting approach to estimate the causal effect of treatment on the outcome. This approach applies a logistic regression model incorporating the confounder as an explanatory variable to estimate an individual's probability of adhering to a particular allocation arm. The inverse of these predicted probabilities are used as weights to inflate or deflate the individual's influence on the overall treatment effect in the arm ^14^.

Lastly, we used instrumental variable estimation in scenarios where non-adherent participants receiving treatment of the opposite arm. This approach analyzes all participants by quantifying first, the degree to which allocated treatment predicts actual treatment and, second the degree to which treatment predicts outcome ^15^. We adopted the structural mean model, first proposed by Robins and Rotnitzky for estimation of the received treatment effect on a dichotomous outcome in randomized trials ^16^. The main assumptions in using instrumental variable estimation are that: i) the instrument, *Z*, is associated with the actual treatment received, *A*; ii) *Z* does not affect the outcome, *Y*, except through its potential effect on A; and iii) *Z* and *Y* do not share causes ^17^. Out of these conditions, only the first is verifiable. In the context of a randomized controlled trial, randomization is an appropriate instrument. When done correctly, randomization satisfies the first and third conditions as it randomly allocates treatment to the participants, independent of the final outcomes. The second condition is satisfied in a successfully double blinded study. When the non-adherence pattern involves switching of treatment to an alternative other than the experimental or control treatment, preference-based analyses using a framework involving ‘compliers’, ‘preferers’ and ‘insisters’ which allows for comparison of treatment effects of two active treatments are available ^18^. However, this involves additional assumptions on the treatment effects in the various arms of participants which are often not verifiable. Details of the analysis methods are provided in *Extended data* (Supplementary 1) ^18^.

### Non-inferiority hypothesis testing

The null hypothesis is tested by comparing the upper bound of the two-sided 95% confidence interval of the effect estimate with the non-inferiority margin. Non-inferiority is concluded if the upper bound of the 95% confidence interval for the absolute risk difference between the experimental and control treatments is less than the non-inferiority margin.

### Simulation mechanism

We generated individual level data which included the following variables: treatment allocation, participant characteristics, which may affect adherence and outcome, actual treatment received, counterfactual outcomes and observed outcomes. Allocation is a binary variable with each individual having a 50% probability of being allocated to the experimental treatment. Participant characteristics were represented by a single continuous variable on the interval [0, 1] drawn from a Beta distribution. This can be thought of as a disease risk score ^19^.

**Figure 1.**
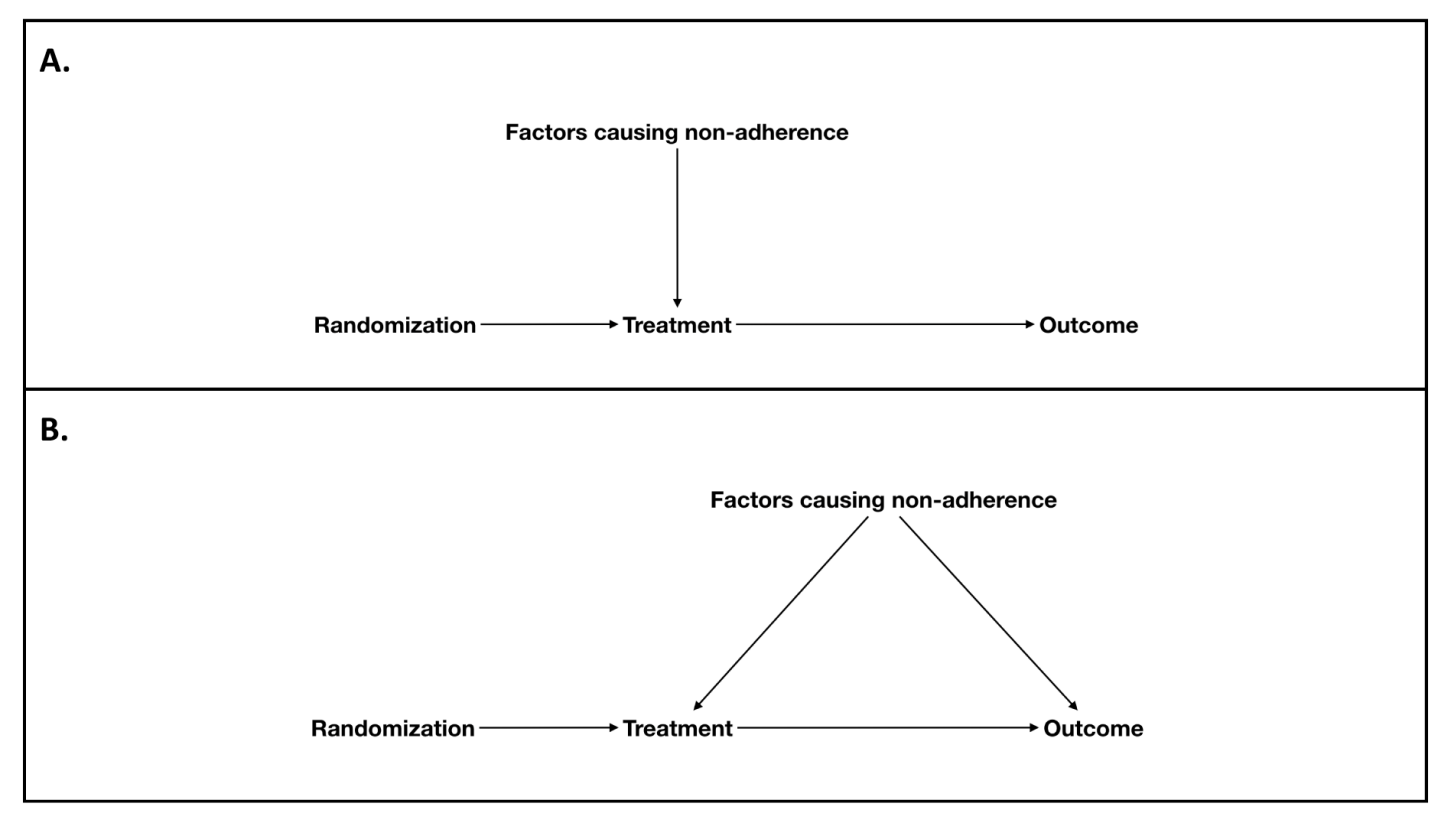
Directed acyclic graphs demonstrating the causal relationships of the variables generated for each study participant. In scenario **A**, factors that cause non-adherence affect the probability of the participant taking up the allocated treatment but do not affect the outcome e.g. minor side effects of the treatment drug. In scenario **B**, factors causing non-adherence affect both the probability of the participant taking up the allocated treatment as well as the outcome e.g. disease severity.

We considered two common reasons for non-adherence. The first is when non-adherence is due to factors which affect the probability of taking up the allocated treatment but do not affect the study outcome through any other pathway ( Figure 1A). The second is driven by confounders, defined as the study participants’ prognostic factors that affect both the probability for taking up the allocated treatment and the outcome ( Figure 1B).

The actual treatment received by an individual differs from the allocated treatment when there is non-adherence. We considered scenarios where non-adherent participants cross over to the opposite treatment arm, or receive alternative treatments that are inferior to both the control and experimental treatments. In the case where the participant characteristics cause an individual to switch to an experimental treatment, their probability for crossing over to the experimental treatment when randomized to the control arm is increased. An example is a trial studying an experimental treatment for a terminal disease which has few effective treatment options. An individual with more severe disease may be more likely to switch to the experimental treatment even when they are randomized to the control treatment. In another case where the factor causing non-adherence discourages an individual to take up an experimental treatment, their probability for adhering to the experimental treatment after being randomized to the experimental arm is decreased. The individual might take up the control treatment or refuse treatment altogether. An example is a trial comparing an experimental exercise regime to nicotine patches for smoking cessation. An individual with chronic obstructive lung disease may be more likely to be non-adherent to the experimental exercise regime and take up nicotine patch or decline all treatments.

Although adherence was considered as a binary variable in the simulations, in the scenario where participants received less effective treatments than the control and experimental treatments may reflect partial adherence to either treatments.

We generated counterfactual outcomes for each individual, one for experimental treatment, one for control treatment and one for alternative treatments inferior to both the control and experimental treatments. The overall average difference between the counterfactual outcomes for experimental and control treatments for all study participants is the pre-defined true treatment effect assumed in the simulations. The participant characteristics may cause an increase or decrease in the probability of having the outcome depending on the direction of influence the confounder has on the outcome. The observed outcome is then chosen from one of the counterfactuals depending on the actual treatment that the individual received. Detailed descriptions of the simulations are included in the supplementary material.

**Figure 2.**
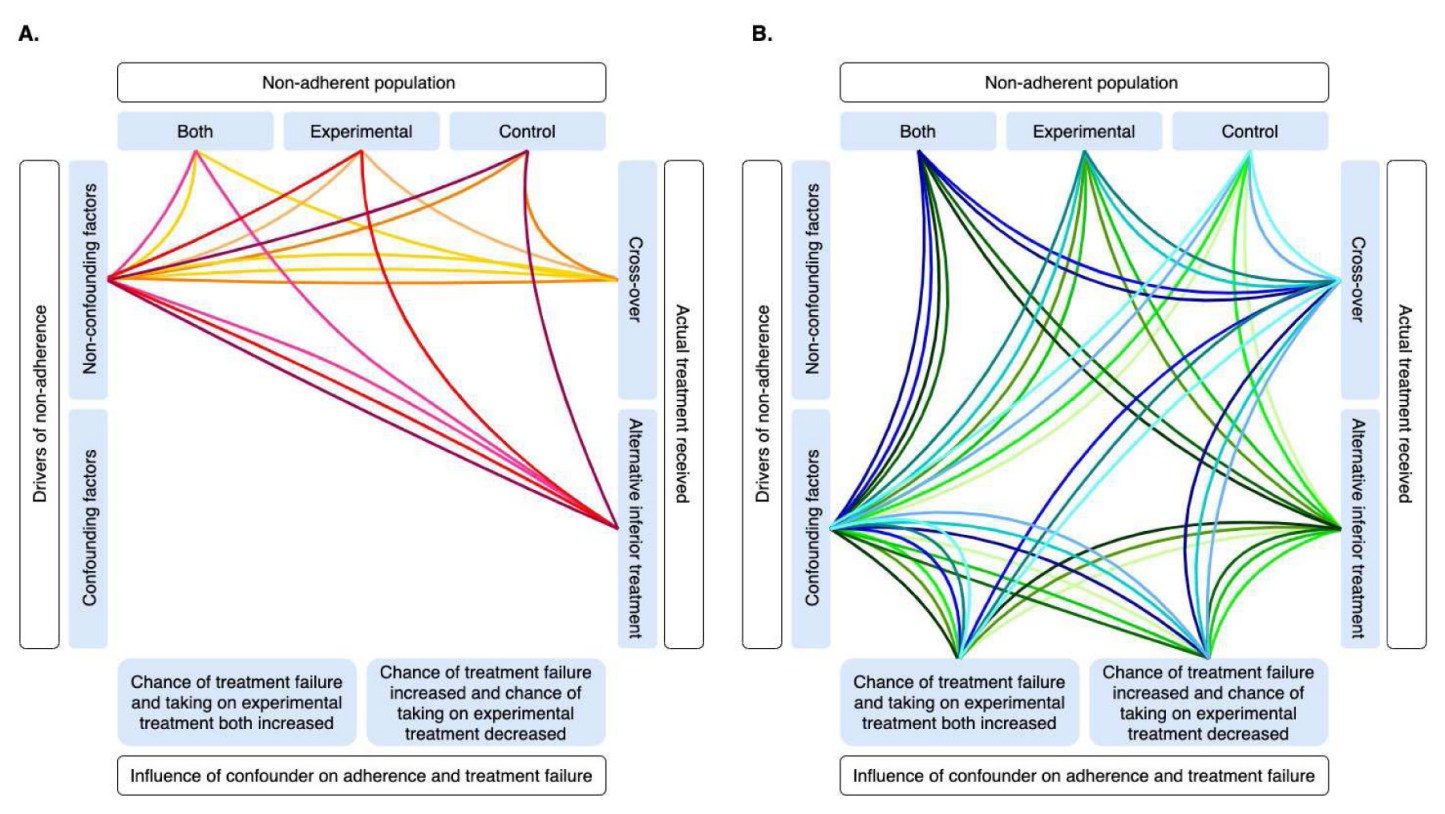
Simulation scenarios. Simulation scenarios were explored permutations of four factors: i) non-adherent population (both arms, experimental arm, control arm); ii) actual treatment received by the non-adherent population (crossing over to the opposite arm, another treatment inferior to both the experimental and control treatment); iii) reason for non-adherence (due to confounding factors or non-confounding factors); iv) if non-adherence is due to non-confounding factors, direction of influence of the confounders on the probability of taking up the experimental treatment and outcome (both probabilities may increase or decrease, or the two probabilities are in opposite directions). Left Panel shows the six possible scenarios when non-adherence is due to non-confounding factors. Right Panel shows the 12 possible scenarios when non-adherence is due to confounding factors. Each coloured line represents one scenario.

We simulated 18 different patterns of non-adherence. The conditions of these non-adherent patterns are shown in Figure 2. Graphs illustrating effect estimates and associated type 1 errors for all simulated scenarios are included in the Supplementary 2 Figure 2.

### Comparing the analysis methods

To examine type 1 error, i.e. concluding non-inferiority when the experimental treatment is actually inferior, we assumed a difference in the probability of treatment failure between the control and experimental arms of 0.1 (i.e. the experimental treatment is inferior and its true treatment effect is 0.1 on an absolute scale). In the case of non-adherent participants receiving an alternative inferior treatment compared to the experimental and control arms, we assumed the difference in the probability of treatment failure between the control and alternative treatment, and experimental and alternative treatment to be 0.1 and 0.2 respectively. Since the non-inferiority margin is assumed to be 10%, simulation iterations which concluded non-inferiority were considered to have committed type 1 error ( *Extended data:* Supplementary 2 Figure 1 ^18^).

Power, given by one minus the type 2 error, is the proportion of non-inferiority trials which conclude non-inferiority correctly. Here, we assumed the true treatment effect to be zero. Thus, the experimental treatment arm has the same probability of having treatment failure i.e. non-inferior to the control treatment. Simulation iterations which concluded inferiority were considered to have committed a type 2 error ( *Extended data:* Supplementary 2 Figure 1 ^18^).

The above assumptions on treatment effects used in calculating type 1 error and power for the scenarios below are arbitrary and intended for illustrative purposes. Other assumptions can be explored with the Shiny app ( https://moru.shinyapps.io/samplesize_nonadherence/).

## Results

### Non-adherent participants receive treatment from the opposite arm

**Figure 3.**
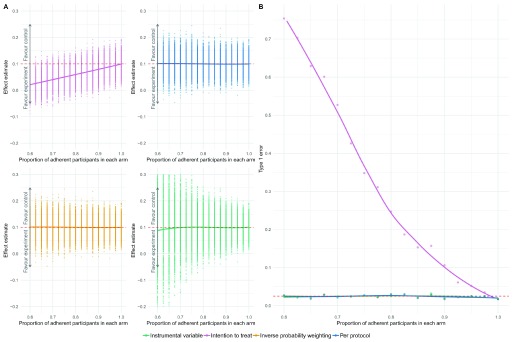
Non-adherence caused by non-confounding factors. **A**: Dots represent trial estimates calculated from each iteration. Coloured lines present the Locally Weighted Scatterplot Smoothing (LOESS) lines through mean trial estimates from all iterations. Because our outcome in the simulated trial refers to treatment failure, higher effect estimate values favour control treatment. The red dotted line is the true effect size estimate assumed in the simulations. **B**: Dots represent type 1 error calculated from all iterations at various degrees of adherence. The tolerable type I error is set at 0.025 at full adherence.

***Non-adherence due to non-confounding factors.*** In most patterns of non-adherence, ITT estimates tend to shift towards zero difference between the control and experimental arms. The only exceptions are when study participants allocated to the experimental arm actually received no treatment or a treatment inferior to both treatments offered in the trial. Compared to treatment efficacy estimates, ITT analysis has a higher tendency of claiming non-inferiority when the experimental treatment is actually inferior when there is non-adherence. Figure 3 illustrates the case where non-adherent study participants cross over to the opposite arm. Even at a relatively high adherence of 90%, the type 1 error of the ITT estimate can be as high as 10%. All other analysis methods are unbiased in this case where non-adherence is due only to non-confounding factors. Note the different scale for the instrumental variable estimates, and the high variance at low adherence.

## Non-adherence due to confounders and no unobserved confounding

**Figure 4.**
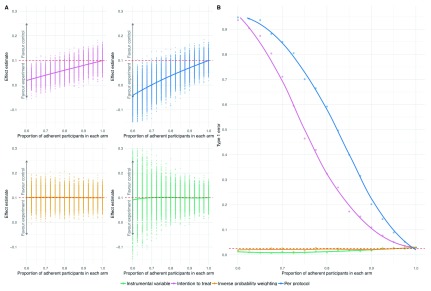
Non-adherence caused by confounding factors I. Non-adherence caused by confounding factors where participants with higher confounder values have lower probability of taking up the allocated treatment regardless of the allocation, and increases the probability of treatment failure.

In the case where confounders influence non-adherence behavior, PP analysis is biased in estimating the causal effect of treatment. Figure 4 illustrates an example where increasing confounder value decreases the probability of taking up the experimental treatment (with a corresponding increase in the probability of taking up the control treatment) and increases the probability of treatment failure. This is such that participants with the highest confounder values in the experimental arm cross over to the control arm and participants with the lowest confounder values in the control arm cross over to the experimental arm. This will lead to an inflated type 1 error rate. In this case, inverse probability weighting and instrumental variable estimation give unbiased estimates with conservative type I error rates.

**Figure 5.**
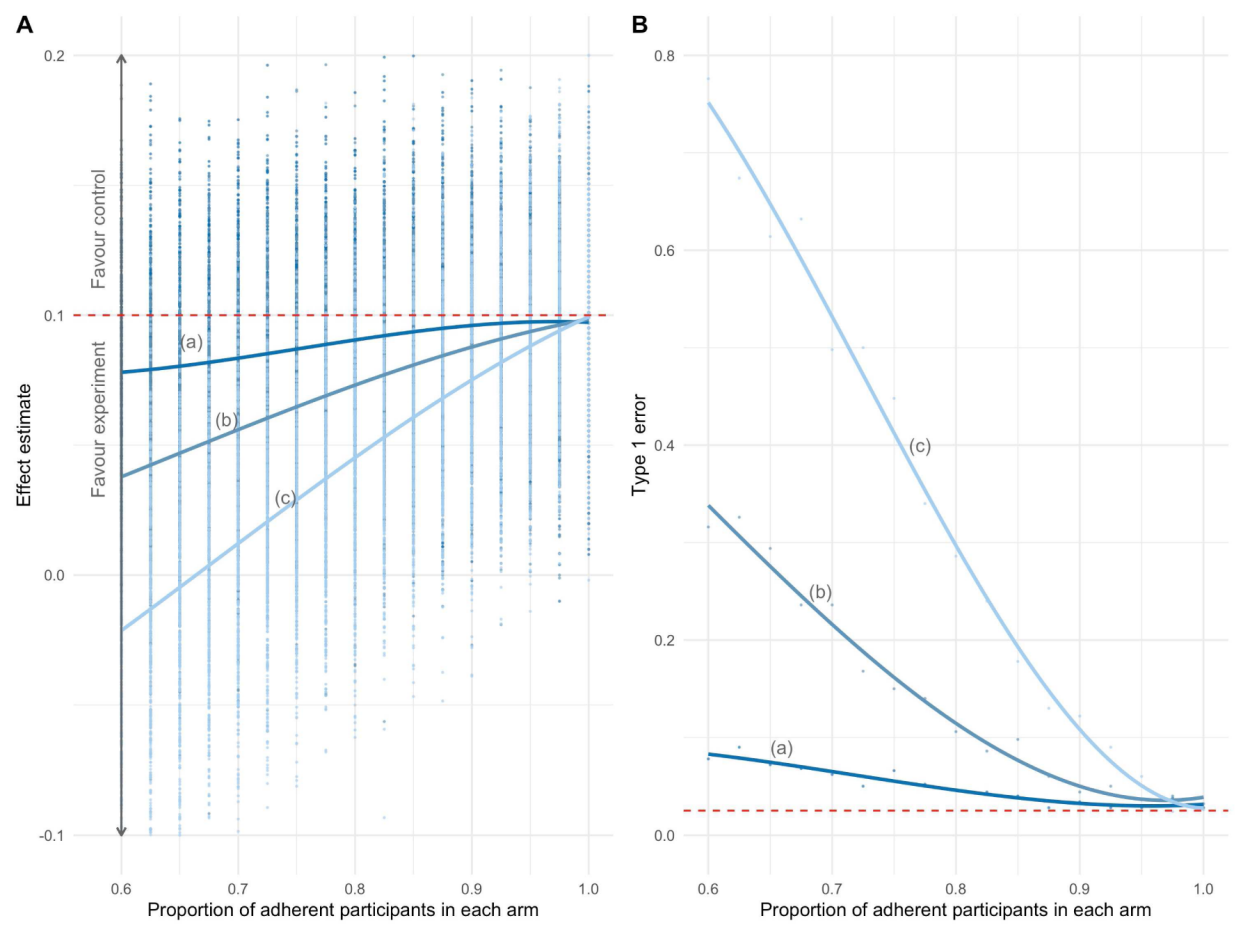
Non-adherence caused by confounding factors II. Non-adherence caused by confounding factors where participants with higher confounder values have lower probability of taking up the experimental treatment regardless of the allocation, and increases the probability of treatment failure. Per protocol analysis is shown (in various shades of blue) to illustrate the impact of increasing direct confounder effect on treatment failure, in terms of the treatment estimates and associated type 1 errors. The magnitude of direct confounder effect on treatment failure is calculated with treatment failure as the dependent variable, and confounder as the independent variable, in a linear regression. ( **a**) magnitude of direct confounder effect on treatment failure = 1; ( **b**) magnitude of direct confounder effect on treatment failure = 5; ( **c**) magnitude of direct confounder effect on treatment failure = 9.

**Figure 6.**
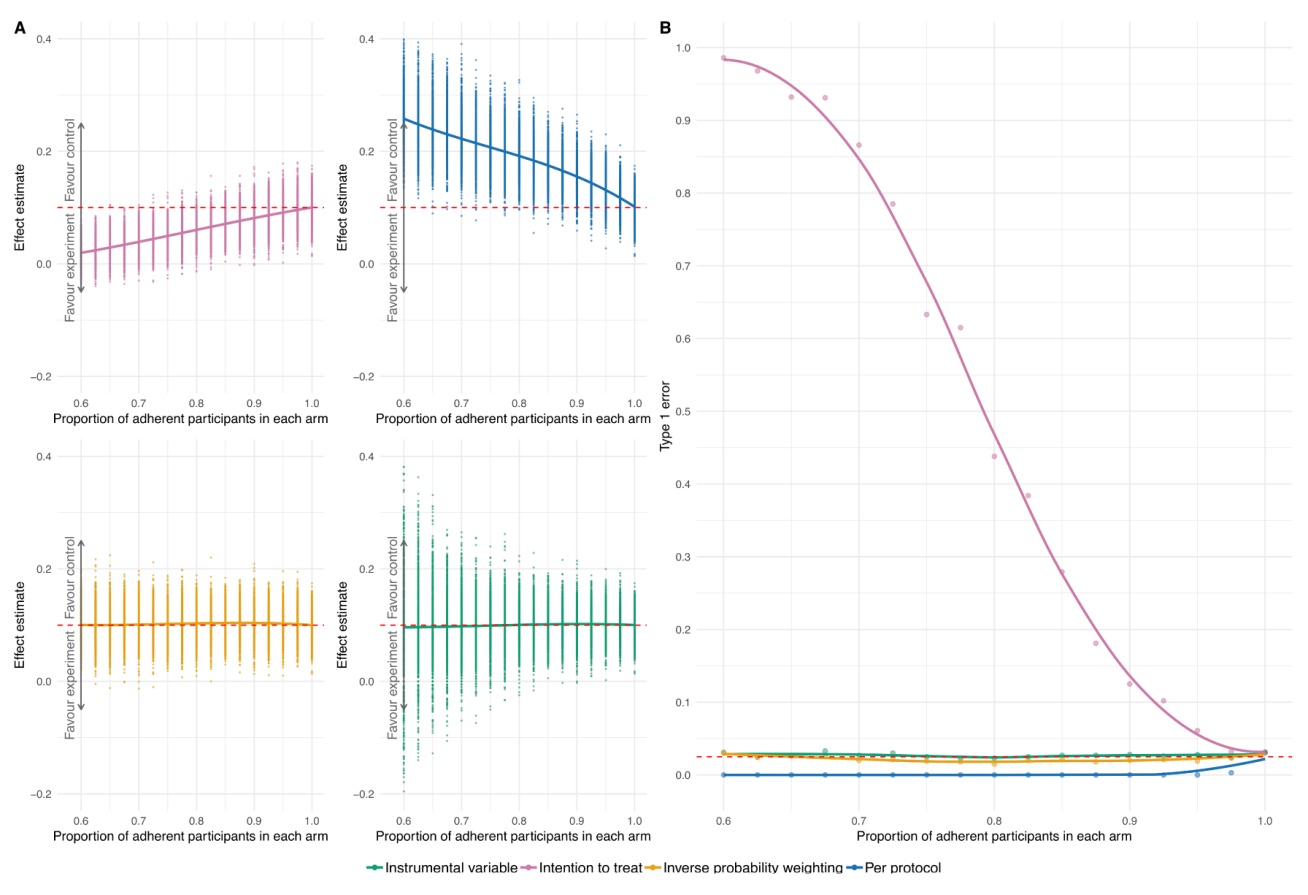
Non-adherence caused by confounding factors III. Non-adherence caused by confounding factors where participants with higher confounder values have higher probabilities of taking up the experimental treatment regardless of the allocation, and treatment failure.

The more influence the confounder has on treatment failure, the more biased PP estimates will be, leading to higher type 1 error rates ( Figure 5). When the confounder increases both the probability of taking up the experimental treatment and of treatment failure, the treatment effect estimated with the PP analysis will be higher than the true value. ( Figure 6)

## Non-adherence due to confounders with unobserved confounding

**Figure 7.**
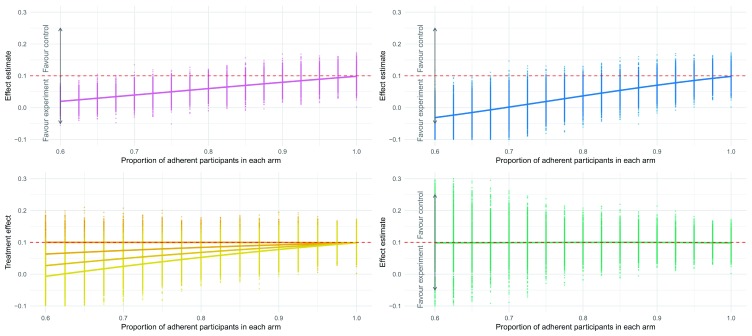
Non-adherence caused by both known and unknown confounders. Four confounders were added in the simulation. ITT, PP and instrumental variable analyses did not adjust for any confounders. For the inverse probability weighting analysis, the four lines represent situations when one, two, three and all of confounders were adjusted for. With more confounders accounted for, treatment estimates become less biased.

In practice, not all confounders will be observed, and those which can be observed may not be measured perfectly so that it will only be possible to partially adjust for confounding. In such cases, inverse probability weighting can become biased ( Figure 7). Adjusting for more confounders can reduce but not eliminate bias in treatment estimates. Instrumental variable estimation, on the other hand, remains unbiased even with unobserved confounders, as it does not depend on the knowledge of the confounders to compute treatment effect estimates when all the above-mentioned assumptions are met.

## Non-adherent participants receive an alternative inferior treatment compared to both the experimental and control treatments

If non-adherent participants do not cross over to the opposite arm, they may receive an alternative inferior treatment or default care. The effect of this on the ITT treatment estimates depends on the allocation arm that is predominantly non-adherent. When most of the non-adherent participants are from the control arm, the control treatment will appear worse compared to the experimental treatment using the ITT analysis, thereby favouring the experimental treatment (Supplementary 2 Figures 2F, 2L, 2R). However, when most of the non-adherent participants are from the experimental arm, the experimental treatment will appear worse compared to the experimental treatment using the ITT analysis, thereby favouring the control treatment (Supplementary 2 Figures 2D, 2J, 2P).

Where non-adherence is caused by confounding factors, PP estimates become biased. The direction of bias is determined by the difference in the underlying prognostic characteristics of the non-adherent participants, similar to the cases where non-adherent participants cross over to the opposite arms.

## Effect of non-adherence on power

In addition to affecting treatment estimates, non-adherence decreases the power to detect truly non-inferior experimental treatments. We consider the effect of non-adherence on inverse probability weighting and instrumental variable effect estimates as these methods can potentially give unbiased treatment efficacy estimates despite non-adherence.

**Figure 8.**
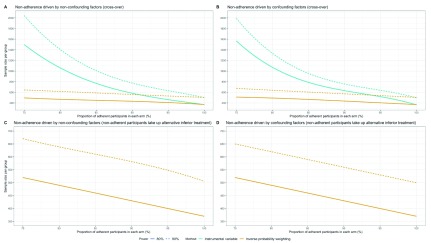
Decrease in adherence requires inflated sample sizes to maintain power. Panel **A** and **C** show the output the scenario where non-adherence is driven by non-confounding factors. Panel **B** and **D** show the output from simulating the scenario where non-adherence is driven by confounding factors. Panels **A** and **B** show the impact of cross-over type of non-adherence on power. The assumed true proportion of treatment failure in the experimental treatment was set to be the same as the control treatment at 40%. Panels **C** and **D** show the impact on power when non-adherent participants receive an alternative treatment inferior to the control and experimental treatments by 10%. In all the four scenarios, the non-inferiority margin was set at 10%.

To maintain power, the sample size required for instrumental variable estimation increases drastically when adherence falls below 95%. In contrast, sample size for inverse probability weighting changes linearly with the decrease in adherence ( Figure 8). In the presence of non-adherence, the more influence the confounder has on treatment failure, the lower the power.

Different patterns of non-adherence and choice of analysis methods affect power to differing degrees. To aid investigators in planning for clinical trials anticipating non-adherence, a power calculator is available online based on the simulation mechanisms shown here ( https://moru.shinyapps.io/samplesize_nonadherence/). Using the same simulation mechanism as above, the calculator caters for a two-arm non-inferiority trial with a binary outcome and time-fixed treatment. The application is an interactive platform that calculates power using user inputs for the following: whether non-adherence is mainly caused by non-confounding and confounding factors; number of participants who are anticipated to be non-adherent; the expected influence the confounder is likely to have on treatment failure; and the various directions of influence the confounders have on adherence and probability of outcomes.

## Discussion

Our simulations illustrate the complexities in interpreting non-inferiority trials with non-adherence, taking both qualitative and quantitative perspectives. Intention-to-treat effect estimates, due to the ‘dilution’ from the participants who received other treatments different from the allocated treatment, tend to be lower than true treatment effects at low adherence under most non-adherence patterns. As non-adherence increases, the chance that ITT analysis will conclude non-inferiority increases. The probability of concluding non-inferiority when the treatment is actually inferior can be increased to as high as 0.1 from the acceptable 0.025 when non-adherence is 90%. The direction of bias in PP analysis is dependent on whether the confounders increase or decrease the probability of taking up the allocated treatment and the probability of the outcome occurring. This bias is increased when the confounder is more influential on the outcome.

Inverse probability weighting accounts for the difference in confounders between the allocation arms to ensure that the reweighted arms are similar and comparable. It eliminates bias if all confounders can be appropriately adjusted for, but in general this will not be possible. Sensitivity analysis methods are available to address unobserved confounding and covariate measurement errors ^20, 21^. In contrast, instrumental variable estimation can account for unknown confounders but requires the "exclusion restriction" to be fulfilled (i.e. treatment allocation only influences the outcome through the treatment and not through any other pathways). This assumption is unverifiable and we are only likely to be confident that it holds in a double blinded study. The other drawback of using an instrumental variable is the need for large sample sizes when adherence is low as the method relies heavily on the strength of the instrument (i.e. randomization) in predicting the treatment. Recent methods using doubly robust procedures have been developed to boost power when using instrumental variable estimation ^22^.

Though our simulation mainly illustrates the analysis of time fixed treatments and outcomes, time varying treatments and outcomes can be analyzed with inverse probability weighting ^23^ and g-estimation methods ^24^. These methods are also used to address missing data and censoring ^25, 26^. Another limitation in our study is that non-adherence is either due to cross-over or switching to a treatment that is inferior to both the control and experimental treatments. In practice, both types of non-adherence may occur within the same trial. However, our simulations use these extreme examples to clarify the impacts of non-adherence on trial analyses and outcomes.

Some degree of non-adherence is near ubiquitous in clinical trials. Though ITT will, under some circumstances, represent the ‘real-world’ effectiveness of treatment allocation, the effects of treatment itself are relevant estimates generalizable to other situations with different adherence patterns. They are also likely to be of particular interest for those with agency in their adherence. When the interest is in the actual treatment effects, as we have shown, the conservative nature of ITT in a conventional superiority trial (i.e. lower probability of concluding superiority in the presence of non-adherence) is compromised under many patterns of non-adherence in a non-inferiority trial.

In conclusion, given the potential inflation in the probability of concluding non-inferiority with non-adherence even in cases where expected non-adherence is as low as 5%, we propose that during the planning stage of clinical trials, investigators should anticipate the likely patterns and magnitude of non-adherence and devise ways to reduce it. Ideally, power calculations should account for such anticipated non-adherence. Potential confounders should be carefully measured and recorded for subsequent analysis. Adjusted analysis of the PP population using inverse probability weighting or g-estimation can reduce bias in treatment effect estimates introduced by non-adherence. In the case of double blinded trials with large sample sizes, instrumental variable estimation may also be appropriate.

## Data availability

### Underlying data

Simulation code is available on GitHub: https://github.com/moyinNUHS/NItrialsimulation.git.

Archived code as at time of publication: https://doi.org/10.5281/zenodo.3746705 ^27^.

License: Creative Commons Zero “No rights reserved” data waiver (CC0 1.0 Public domain dedication).

### Extended data

Zenodo: Statistical considerations in the design and analysis of non-inferiority trials with binary endpoints in the presence of non-adherence: a simulation study (Supplementary material), http://doi.org/10.5281/zenodo.3746706 ^18^.

This project contains the following extended data:

- Supplementary material 1: Simulation models and analysis methods.
- Supplementary material 2: Supplementary figures.

Data are available under the terms of the Creative Commons Zero "No rights reserved" data waiver (CC0 1.0 Public domain dedication).

## Software availability

Power calculator accounting for non-adherence in a non-inferiority trial: https://moru.shinyapps.io/samplesize_nonadherence/
