# Supplementary material for: Statistical considerations in the design and analysis of non-inferiority trials with binary endpoints in the presence of non-adherence: a simulation study
Source: Wellcome Open Res. 2020 Apr 24;4:207. Originally published 2019 Dec 18. [Version 2] doi: 10.12688/wellcomeopenres.15636.2 (PMC7205408; doi:10.12688/wellcomeopenres.15636.2)
Supplement: Peer review history

# Peer review: Reviewer response for version 2

**DOI:** 10.21956/wellcomeopenres.17386.r38525

**Published:** 2020-05-06

**Authors:** Mimi Y. Kim

**Affiliations:** 1 Department of Epidemiology and Population Health, Albert Einstein College of Medicine, Bronx, NY, USA

I very much appreciate the authors' thorough responses to my comments and the revisions to the paper appear appropriate.

Is the rationale for developing the new method (or application) clearly explained?

Yes

Is the description of the method technically sound?

Partly

Are the conclusions about the method and its performance adequately supported by the findings presented in the article?

Partly

If any results are presented, are all the source data underlying the results available to ensure full reproducibility?

Yes

Are sufficient details provided to allow replication of the method development and its use by others?

Partly

Reviewer Expertise:

biostatistics, clinical trials

I confirm that I have read this submission and believe that I have an appropriate level of expertise to confirm that it is of an acceptable scientific standard.

# Peer review: Reviewer response for version 2

**DOI:** 10.21956/wellcomeopenres.17386.r38526

**Published:** 2020-05-04

**Authors:** Matteo Quartagno

**Affiliations:** 1 MRC Clinical Trials Unit, Institute of Clinical Trials and Methodology, University College London, London, UK

Thanks for addressing my previous comments. I have no additional comments.

Is the rationale for developing the new method (or application) clearly explained?

Yes

Is the description of the method technically sound?

Yes

Are the conclusions about the method and its performance adequately supported by the findings presented in the article?

Partly

If any results are presented, are all the source data underlying the results available to ensure full reproducibility?

Yes

Are sufficient details provided to allow replication of the method development and its use by others?

Yes

Reviewer Expertise:

Clinical Trials, Statistics.

I confirm that I have read this submission and believe that I have an appropriate level of expertise to confirm that it is of an acceptable scientific standard.

# Peer review: Reviewer response for version 1

**DOI:** 10.21956/wellcomeopenres.17132.r37977

**Published:** 2020-02-26

**Authors:** Matteo Quartagno

**Affiliations:** 1 MRC Clinical Trials Unit, Institute of Clinical Trials and Methodology, University College London, London, UK

**Summary:** This is a well-planned and well-written paper, explaining some known, yet poorly handled, issues in non-inferiority trials affected by non-adherence. The paper provides results of simulations, and I particularly enjoyed how simple and effective was the presentation of results in graphs. Conclusions of the paper are also reasonable. I only have a few minor comments that I believe might improve the paper:

**Comments: **

1. I think one important point that you should try to stress more, is that you only considered non-adherence to one of the two arms. Potentially, in non-inferiority trials both arms could suffer from non-adherence, as patients could also stop getting active control treatment. This is important to note particularly because non-adherence in both arms could complicate the implementation of Instrumental Variables methods. It would be good to mention the general problem somewhere at the beginning of the paper and the specific issue with IV in the discussion.
2. Similarly, here you only considered binary non-adherence. In reality, patients might get treatment only for some time, then switch, then maybe switch back, etc etc. Of course, I agree it was a good idea to start from a simpler example, but I still think it would be good to both (i) clarify that this is the case and possibly (ii) justify your example a bit better. Your example is a duration reduction trial, so isn’t it the typical situation where it is difficult to think of adherence in binary terms? Could you explain in words exactly what would be adherence or non-adherence in that hypothetical trial?
3. Please clarify across the paper, e.g. when you say that significance level was 0.025 in the methods section, that you used one-sided tests. In section “Non-inferiority hypothesis testing”, it should be 97.5% one-sided or 95% two-sided (in your examples).
4. In Figure 4, it seems like with no non-adherence, type 1 error is well below the expected 2.5%. With 1000 simulations, this seems well outside the Monte-Carlo confidence interval. Could you please (i) check that there is nothing wrong with these simulations and (ii) add to the plot the two lines for the Monte-Carlo confidence interval, so that one can immediately check whether results are outside regular random variation. This is partly the case also for Figure 5.
5. In the final recommendations, you say that researchers should consider consequences of non-adherence if they expect at least 5% non adherence. I personally believe it is always better not to give specific numbers, as these could be successively used to justify choices without much critical thinking. I would rather prefer a more general sentence like "especially those who expect some degree of non-adherence".  

**Typos / formatting / plots:**

1. Reference for the FDA guidelines: FDA considered like a given name, please fix.
2. Sometimes you put citations at the end of a sentence, rather than next to the name of the author. See, e.g., citation 7 and 8, page 3, left column. I’d personally prefer this latter citation format, but feel free to ignore, particularly if this is journal requirement.
3. Page 4, last paragraph of left column, you wrote: *This approach analyzes all participants by quantifying first, the degree to which allocated treatment predicts actual treatment and, second, the degree to which treatment predicts outcome.*
  Please fix punctuation.
4. I like a lot how you visually presented results of simulations. When it comes to methods, though, I would personally prefer to have a simple table of all the simulation scenarios you explored. To me, Figure 2 looks much more convoluted than a simple Table where different dimensions were columns and different scenarios rows. Could you provide that sort of table at least as additional online material?

Is the rationale for developing the new method (or application) clearly explained?

Yes

Is the description of the method technically sound?

Yes

Are the conclusions about the method and its performance adequately supported by the findings presented in the article?

Partly

If any results are presented, are all the source data underlying the results available to ensure full reproducibility?

Yes

Are sufficient details provided to allow replication of the method development and its use by others?

Yes

Reviewer Expertise:

Clinical Trials, Statistics.

I confirm that I have read this submission and believe that I have an appropriate level of expertise to confirm that it is of an acceptable scientific standard.

# Response

**Published:** 2020-04-09

**Authors:** Yin Mo

**Affiliations:** University of Oxford, UK

Dear Dr Quartagno, 

Thank you very much for your detailed review and very thoughtful comments. We hope to have addressed all of your comments in version 2 of the manuscript. 

***1. I think one important point that you should try to stress more, is that you only considered non-adherence to one of the two arms. Potentially, in non-inferiority trials both arms could suffer from non-adherence, as patients could also stop getting active control treatment. This is important to note particularly because non-adherence in both arms could complicate the implementation of Instrumental Variables methods. It would be good to mention the general problem somewhere at the beginning of the paper and the specific issue with IV in the discussion.***

Apologies for not being clear in the manuscript that our simulations do address non-adherence in one arm and both arms. We have clarified this in all the graphs by relabelling the x-axes: “Proportion of adherent participants in each arm”. In the new supplementary 2, we include figures from all simulated scenarios, including those where participants from one arm are non-adherent and those where participants from both arms are not adherent. 

***2. Similarly, here you only considered binary non-adherence. In reality, patients might get treatment only for some time, then switch, then maybe switch back, etc etc. Of course, I agree it was a good idea to start from a simpler example, but I still think it would be good to both (i) clarify that this is the case and possibly (ii) justify your example a bit better. Your example is a duration reduction trial, so isn’t it the typical situation where it is difficult to think of adherence in binary terms? Could you explain in words exactly what would be adherence or non-adherence in that hypothetical trial?***

We agree that this may be a limitation in this simulation study as it is only applied to time-fixed interventions. We have added in the methodology: “Although adherence was considered as a binary variable in the simulations, in the scenario where participants received less effective treatments than the control and experimental treatments may reflect partial adherence to either treatments.” To further explain why in an antibiotic duration trial, adherence is often considered binary, we have added: “With this single end-point, we consider adherence as a binary variable where non-adherent patients in the short arm would receive longer than five days of treatment, and non-adherent patients in the long arm would receive than less than five days of treatment.”

***3. Please clarify across the paper, e.g. when you say that significance level was 0.025 in the methods section, that you used one-sided tests. In section “Non-inferiority hypothesis testing”, it should be 97.5% one-sided or 95% two-sided (in your examples).***

We agree with this recommendation and have clarified in the methodology section that “The null hypothesis is tested by comparing the upper bound of the two-sided 95% confidence interval of the effect estimate with the non-inferiority margin.”

***4. In Figure 4, it seems like with no non-adherence, type 1 error is well below the expected 2.5%. With 1000 simulations, this seems well outside the Monte-Carlo confidence interval. Could you please (i) check that there is nothing wrong with these simulations and (ii) add to the plot the two lines for the Monte-Carlo confidence interval, so that one can immediately check whether results are outside regular random variation. This is partly the case also for Figure 5.***

We have changed the way we derive the standard errors of the simulated treatment estimates from using standard formulae to empirically from the simulated distributions. We have updated these graphs showing type 1 error rates close to the nominal 0.025 level. We did not include the Monte-Carlo confidence intervals as the type 1 error rates now fall nicely on the expected value of 0.025, and adding further shadings and lines for confidence intervals may reduce the prominence of the lines formed by estimates.

***5. In the final recommendations, you say that researchers should consider consequences of non-adherence if they expect at least 5% non adherence. I personally believe it is always better not to give specific numbers, as these could be successively used to justify choices without much critical thinking. I would rather prefer a more general sentence like "especially those who expect some degree of non-adherence".***

We agree with this concern, and have changed this sentence to “In conclusion, given the potential inflation in the probability of concluding non-inferiority with non-adherence even in cases where expected non-adherence is as low as 5%, we propose that during the planning stage of clinical trials investigators should anticipate the likely patterns and magnitude of non-adherence and devise ways to reduce it.”

***6. Reference for the FDA guidelines: FDA considered like a given name, please fix.***

We have amended the reference to “Center for Drug Evaluation and Research ( Food and Drug Administration). Non-inferiority clinical trials to establish effectiveness: guidance for industry 2016. Accessed Oct 2017;6.” in version 2 of the manuscript. 

***7. Sometimes you put citations at the end of a sentence, rather than next to the name of the author. See, e.g., citation 7 and 8, page 3, left column. I’d personally prefer this latter citation format, but feel free to ignore, particularly if this is journal requirement.***

We will liaise with the journal editors to ensure the format of the references are correct. 

***8. Page 4, last paragraph of left column, you wrote: This approach analyzes all participants by quantifying first, the degree to which allocated treatment predicts actual treatment and, second, the degree to which treatment predicts outcome. Please fix punctuation.***

We have removed the comma after ‘second’.

***9. I like a lot how you visually presented results of simulations. When it comes to methods, though, I would personally prefer to have a simple table of all the simulation scenarios you explored. To me, Figure 2 looks much more convoluted than a simple Table where different dimensions were columns and different scenarios rows. Could you provide that sort of table at least as additional online material?***

We have now included this table in supplementary 2 with all the simulation scenarios included.

Please do not hesitate to contact us again for further comments. Once again, we would like to express our gratitude towards your insightful comments! 

Yours sincerely, 

Mo Yin

# Peer review: Reviewer response for version 1

**DOI:** 10.21956/wellcomeopenres.17132.r37440

**Published:** 2020-01-13

**Authors:** Mimi Y. Kim

**Affiliations:** 1 Department of Epidemiology and Population Health, Albert Einstein College of Medicine, Bronx, NY, USA

The authors have conducted extensive simulations to evaluate the impact of various patterns of non-adherence on the treatment effect estimates, Type 1 error rate and power of different methods for analyzing non-inferiority trials.Many of the key findings, particularly those pertaining to the ITT, per-protocol, and instrumental variables methods, have been reported by others. What is novel in this paper is that potential confounders of non-adherence are considered, and a user-friendly app was developed that allows one to evaluate the impact on power of different degrees of non-adherence.The paper could be improved, however, by resolving several inconsistencies and errors in notation, and by providing additional details about how the simulations were performed. Below are some specific comments:

  

- It should be explicitly stated in the paper that in the simulation studies, it was assumed that non-adherence is all or nothing (i.e., no partial non-adherence). Also, when non-adherence does occur, it is assumed to be either always cross-over, or always switching to an alternative that is inferior to both experimental and control treatments. The authors may want to comment on whether this second set of assumptions is very realistic since in a non-inferiority trial with an active control arm, one would expect that both cross-over and switching to an inferior alternative would occur in the same study. The smoking cessation trial described in the paper is actually an example where both could happen since an individual assigned to the experimental exercise regimen may not adhere by either crossing over and taking up the nicotine patch (control) or switching to the inferior alternative of declining all treatments.
-  It is unclear from the description of the simulations how the impact of switching to an alternative treatment that is inferior to both the experimental or control treatment was evaluated. Step 4 in the supplementary material addresses only the cross-over non-adherence pattern (A= 0, 1).  Also, the assumed probability of failure on this inferior alternative was not stated and would presumably have a major impact on the ITT results. 
- More detailed results should be provided for the non-adherence pattern where subjects could receive no treatment or one that is inferior to the trial treatments.  Figure 3 seems to address only the cross-over case.
- Page 5: “… *when the experimental treatment is actually inferior*, *we assumed a difference in the probability of treatment failure between the control and experimental arms of 0.1 (i.e. the experimental treatment is inferior and its true treatment effect is -0.1 on an absolute scale)*.”  The outcome was defined on Page 3 as treatment failure (Y=1 represents treatment failure and Y=0 represents treatment success). Since outcome rates that are higher in the experimental compared to the control arm are in the direction of inferiority, wouldn’t it be more accurate to re-state the above sentence as “… *we assumed a difference in the probability of treatment failure between the experimental and controls arms of 0.1 (i.e. the experimental treatment is inferior and its true treatment effect is 0.1 on an absolute scale)*.” 
- It is difficult to identify from the colored lines in Figure 2 which six scenarios in the left panel and which twelve scenarios in the right panel were considered in the simulation studies. It would help if different line patterns in addition to colors were used to denote the various scenarios.
- Figure 2 suggests that the simulations considered scenarios where non-adherence occurs in both groups, experimental group only or control group only, so it is not clear in all the X-axes that are labeled as “proportion of adherent participants”, which group(s) this proportion refers to. Is the proportion of adherent participants assumed to be the same in the both treatment groups?
- Page 6 states that “ *Figure 4 illustrates an example where increasing confounder value decreases the probability of taking up the experimental treatment*”. Is this confounding relationship assumed only in the experimental arm? What non-compliance pattern/rate is assumed in the control arm? Or did the authors mean they assumed increasing confounding value decreases the probability of adherence/taking up the allocated treatment in both arms as suggested by the Figure 4 title? The right and left panels of Figure 2 also state “ *Chance of treatment failure and taking up experimental treatment both increased or decreased*.” Please clarify if this is supposed to be experimental treatment (in experimental arm only?) or allocated treatment (in both arms?).
- The app which allows one to explore the impact of non-adherence in a non-inferiority trial is potentially useful, but it seems it is only applicable to the case where cross-over is the only form of non-compliance. This should be clearly stated. Also, in the case of confounding factors, one needs to specify the “ *effect of confounder on taking up the experimental treatment*”. Again, it should be clarified whether the authors indeed mean the effect of confounder on taking the experimental treatment in the experimental group only or taking the assigned treatment (whichever group the participant was assigned to). The supplementary material (Step 2) and the titles for Figures 5 and 7 suggest the latter (Figure 5: “ … *higher confounder value decreases the probability of taking up the allocated treatment*”).
- With the authors’ approach for evaluating the impact of potential confounders, one does not have a clear sense for exactly how much confounding is occurring since only the direction of the effect of the confounder on the adherence and treatment failure probabilities is specified and not the magnitude of the confounding effect. It would be informative if, for a few of the confounding scenarios, the mean estimated failure rates (from all simulation iterations) in the non-adherers could be contrasted with the corresponding mean failure rate among adherers (separately in the experimental and control arms) so readers have a more intuitive idea of the degree of selection bias that is occurring due to the specific way that confounding was generated in the simulations.
- It is not clear why the Type 1 error rates for the IV and IPP methods are below the nominal 0.025 level in Figures 4 and 5.
- Page 4, line 10: there in an error in the second term of the expression for the PP effect.
- Page 4, Non-inferiority hypothesis testing: it should be clarified that the upper bound of the 95% CI for the absolute risk difference (experimental – control) needs to be less than the NI margin.

Is the rationale for developing the new method (or application) clearly explained?

Yes

Is the description of the method technically sound?

Partly

Are the conclusions about the method and its performance adequately supported by the findings presented in the article?

Partly

If any results are presented, are all the source data underlying the results available to ensure full reproducibility?

Yes

Are sufficient details provided to allow replication of the method development and its use by others?

Partly

Reviewer Expertise:

biostatistics, clinical trials

I confirm that I have read this submission and believe that I have an appropriate level of expertise to confirm that it is of an acceptable scientific standard, however I have significant reservations, as outlined above.

# Response

**Published:** 2020-04-09

**Authors:** Yin Mo

**Affiliations:** University of Oxford, UK

Dear Professor Kim, 

Thank you very much for your detailed review and very thoughtful comments. We hope to have addressed all of your comments in version 2 of the manuscript. 

Below we describe the specific improvements and corrections we made according to your recommendations:

***1. It should be explicitly stated in the paper that in the simulation studies, it was assumed that non-adherence is all or nothing (i.e., no partial non-adherence). Also, when non-adherence does occur, it is assumed to be either always cross-over, or always switching to an alternative that is inferior to both experimental and control treatments. The authors may want to comment on whether this second set of assumptions is very realistic since in a non-inferiority trial with an active control arm, one would expect that both cross-over and switching to an inferior alternative would occur in the same study. The smoking cessation trial described in the paper is actually an example where both could happen since an individual assigned to the experimental exercise regimen may not adhere by either crossing over and taking up the nicotine patch (control) or switching to the inferior alternative of declining all treatments.***

We agree that a limitation of the simulations is that non-adherence is either due to cross-over or switching to an alternative treatment that is inferior to both the control and experimental treatments. We have acknowledged this in the discussions by adding “Another limitation in our study is that non-adherence is either due to cross-over or switching to a treatment that is inferior to both the control and experimental treatments. In practice, both types of non-adherence may occur within the same trial. However, our simulations use these extreme examples to clarify the impacts of non-adherence on trial analyses and outcomes.” We have also acknowledged in the manuscript that non-adherence is a binary variable in the simulations. 

***2. It is unclear from the description of the simulations how the impact of switching to an alternative treatment that is inferior to both the experimental or control treatment was evaluated. Step 4 in the supplementary material addresses only the cross-over non-adherence pattern (A= 0, 1).  Also, the assumed probability of failure on this inferior alternative was not stated and would presumably have a major impact on the ITT results.***

Thank you for this valuable feedback. We have updated the methodology sections in both the manuscript and the supplementary material to include the case where non-adherent participants receive an alternative treatment inferior to both the control and experimental treatments. The presumed probability of failure for the inferior alternative treatment is also included in the methodology section in version 2 of the main manuscript.

***3. More detailed results should be provided for the non-adherence pattern where subjects could receive no treatment or one that is inferior to the trial treatments. Figure 3 seems to address only the cross-over case.***

We have added descriptions of our key findings from the scenarios where subjects could receive no treatment or one that is inferior to the trial treatments in the results section. We have also included all 18 simulation scenarios in version 2 of the supplementary material 2.

***4. Page 5: “…when the experimental treatment is actually inferior, we assumed a difference in the probability of treatment failure between the control and experimental arms of 0.1 (i.e. the experimental treatment is inferior and its true treatment effect is -0.1 on an absolute scale).”  The outcome was defined on Page 3 as treatment failure (Y=1 represents treatment failure and Y=0 represents treatment success). Since outcome rates that are higher in the experimental compared to the control arm are in the direction of inferiority, wouldn’t it be more accurate to re-state the above sentence as “…we assumed a difference in the probability of treatment failure between the experimental and controls arms of 0.1 (i.e. the experimental treatment is inferior and its true treatment effect is 0.1 on an absolute scale).”***

We have amended this error i.e. changed -0.1 to 0.1. 

***5. It is difficult to identify from the colored lines in Figure 2 which six scenarios in the left panel and which twelve scenarios in the right panel were considered in the simulation studies. It would help if different line patterns in addition to colors were used to denote the various scenarios.***

***All the scenarios are explored in the simulations. This includes 6 on the left panel (non-adherence driven by non-confounding factors) and 12 on the right panel (non-adherence driven by confounding factors.***

To improve clarity, we have included graphs produced from all the scenarios in the version 2 of the supplementary material 2.

***6. Figure 2 suggests that the simulations considered scenarios where non-adherence occurs in both groups, experimental group only or control group only, so it is not clear in all the X-axes that are labeled as “proportion of adherent participants”, which group(s) this proportion refers to. Is the proportion of adherent participants assumed to be the same in the both treatment groups?***

We have changed the x-axis label from ‘proportion of adherent participants’ to ‘proportion of adherent participants in each arm’ such that the proportion of adherent participants is the same in both groups. 

***7. Page 6 states that “Figure 4 illustrates an example where increasing confounder value decreases the probability of taking up the experimental treatment”. Is this confounding relationship assumed only in the experimental arm? What non-compliance pattern/rate is assumed in the control arm? Or did the authors mean they assumed increasing confounding value decreases the probability of adherence/taking up the allocated treatment in both arms as suggested by the Figure 4 title? The right and left panels of Figure 2 also state “Chance of treatment failure and taking up experimental treatment both increased or decreased.” Please clarify if this is supposed to be experimental treatment (in experimental arm only?) or allocated treatment (in both arms?).***

The effect of the confounder (increasing confounder value decreases the probability of taking up the experimental treatment) has a corresponding increase in the probability of taking up the control treatment. We have added ‘This is such that participants with the highest confounder values in the experimental arm cross over to the control arm, and participants with the lowest confounder values in the control arm cross over to the experimental arm.’ to further explain this point. We have also changed the captions of Figure 4 and 5 to “participants with higher confounder values have lower/higher probability of taking up the experimental treatment regardless of the allocation, and lower/higher probability of treatment failure” improve clarity. 

***8. The app which allows one to explore the impact of non-adherence in a non-inferiority trial is potentially useful, but it seems it is only applicable to the case where cross-over is the only form of non-compliance. This should be clearly stated. Also, in the case of confounding factors, one needs to specify the “effect of confounder on taking up the experimental treatment”. Again, it should be clarified whether the authors indeed mean the effect of confounder on taking the experimental treatment in the experimental group only or taking the assigned treatment (whichever group the participant was assigned to). The supplementary material (Step 2) and the titles for Figures 5 and 7 suggest the latter (Figure 5: “ …higher confounder value decreases the probability of taking up the allocated treatment”).***

We have added to our shiny app an additional input that includes the scenario where non-adherent participants take up an alternative treatment. We have also clarified the effect of the confounder on the shiny app by elaborating on the tabs: “Participants with high confounder values from the standard-of-care arm tend to be non-adherent; participants with low confounder values from from the experimental arm tend to be non-adherent" and "Participants with low confounder values from the standard-of-care arm tend to be non-adherent; participants with high confounder values from from the experimental arm tend to be non-adherent”.

***9. With the authors’ approach for evaluating the impact of potential confounders, one does not have a clear sense for exactly how much confounding is occurring since only the direction of the effect of the confounder on the adherence and treatment failure probabilities is specified and not the magnitude of the confounding effect. It would be informative if, for a few of the confounding scenarios, the mean estimated failure rates (from all simulation iterations) in the non-adherers could be contrasted with the corresponding mean failure rate among adherers (separately in the experimental and control arms) so readers have a more intuitive idea of the degree of selection bias that is occurring due to the specific way that confounding was generated in the simulations.***

We have added an additional figure (figure 5) to highlight that the more influence the confounder has on treatment failure, the more biased per protocol estimates will be, leading to higher type 1 error rates. 

***10. It is not clear why the Type 1 error rates for the IV and IPP methods are below the nominal 0.025 level in Figures 4 and 5.***

We have changed the way we derive the standard errors of the simulated treatment estimates from using standard formulae to empirically from the simulated distributions. We have updated these graphs showing type 1 error rates close to the nominal 0.025 level. 

***11. Page 4, line 10: there in an error in the second term of the expression for the PP effect.***

Thank you for pointing out his typo. This notation error has been corrected from Pr[Y ^A=1, Z=1^ =1] -  Pr[Y ^A=1, Z=1^ =1] to Pr[Y ^A=1, Z=1^ =1] -  Pr[Y ^A=0, Z=0^ =1].

***12. Page 4, Non-inferiority hypothesis testing: it should be clarified that the upper bound of the 95% CI for the absolute risk difference (experimental – control) needs to be less than the NI margin.***

We have included “Non-inferiority is concluded if the upper bound of the 95% confidence interval for the absolute risk difference between the experimental and control treatments is less than the non-inferiority margin.” in the non-inferiority hypothesis testing.

Once again, we thank you for your comments and please do not hesitate to raise further queries! 

Your sincerely, 

Mo Yin
